# Fabry disease and incidence of cancer

**DOI:** 10.1186/s13023-017-0701-6

**Published:** 2017-09-06

**Authors:** Sarah Bird, Efthymios Hadjimichael, Atul Mehta, Uma Ramaswami, Derralynn Hughes

**Affiliations:** 1 Royal Free London NHS Foundation Trust, London, UK; 2 Nottingham University Hospitals NHS Trust, Nottingham, UK

**Keywords:** Fabry disease, Lysosomal storage disorders, Haematology, Genetics, Cancer: Urological, Cancer: Dermatological, Cancer: CNS

## Abstract

**Background:**

Fabry disease is an X-linked lysosomal storage disorder caused by deficient activity of *α*-galactosidase A and the resulting accumulation of the glycosphingolipid globotriaosylceramide (Gb3) and its derivatives, including globotriaosylsphingosine (Lyso-Gb3). Increased cellular and plasma levels of Gb3 and Lyso-Gb3 affect multiple organs, with specific clinical consequences for the kidneys, heart and brain.

There is growing evidence that alterations in glycosphingolipids may have an oncogenic role and this prompted a review of cases of cancer and benign lesions in a large single centre cohort of Fabry patients. We also explored whether there is a difference in the risk of cancer in Fabry patients compared to the general population.

**Results:**

Our results suggest that Fabry patients may have a marginally reduced rate of all cancer (incidence rate ratio 0.61, 95% confidence interval 0.37 to 0.99) but possibly increased rates of melanoma, urological malignancies and meningiomas.

**Conclusion:**

Greater knowledge and awareness of cancer in patients with Fabry disease may help identify at-risk individuals and elucidate cancer mechanisms in this rare inherited disease, which may potentially be relevant to the wider cancer population.

## Background

Fabry disease, one of the most prevalent lysosomal storage disorders (LSD), is caused by mutations in the *GLA* gene which leads to partial or complete deficiency of the lysosomal enzyme *α*-galactosidase A (AGAL A, OMIM *300644). Over 800 different mutations have been recorded in Fabry patients, including a variety of missense or nonsense point mutations, splicing mutations, deletions and insertions [1]. Although the inheritance of Fabry disease is X-linked, up to 70% of heterozygote females are symptomatic, with some experiencing severe disease manifestations, similar to males [1, 2]. The mechanism behind this may in part be skewed X-inactivation but this is an area of debate [3, 4].

Reduced or absent activity of AGAL A results in accumulation of globotriaosylceramide (Gb3) and its derivatives, including globotriaosylsphingosine (lyso-Gb3), in plasma and cells throughout the body [5]. Clinical features include neuropathic pain, characteristic angiokeratoma, gastrointestinal symptoms and fatigue, and ultimately renal failure, cardiomyopathy and stroke [6]. Two broad clinical phenotypes are recognised (although there is likely to be a continuum); an early onset classical form exhibiting pain, angiokeratoma and sweating abnormalities preceding overt kidney and heart disease, and a later onset form with predominant manifestation in a single organ, usually the heart [6].

Therapy is essentially replacement of the deficient enzyme by intravenous enzyme replacement therapy (agalsidase alfa (Shire), agalsidase beta (Genzyme Sanofi)) or oral pharmacological chaperone therapy (Migalastat (Amicus therapeutics)). Gene therapy is also in development [7].

The pathophysiological link between accumulation of Gb3/Lyso-Gb3 and organ pathology, such as fibrosis, is not well understood. Substrate accumulation may lead to ischemia and cell death or initiate other downstream processes including inflammation, apoptosis or formation of reactive oxygen species [8]. Glycosphingolipids (GSL) have themselves been implicated in both oncogenesis and potential cancer therapies [9]. They are an integral part of the cell membrane and exhibit heterogeneous glycosylation and ceramide structures, thereby functioning as antigens, mediators of cell adhesion and modulators of signal transduction [10]. Specific GSLs may be highly expressed in tumour cells and act as adhesion molecules in tumour cell metastasis and modulators of tumor growth [10].

Elevated expression of Gb3 has been identified in multiple cancer types, including breast, colon, pancreatic, gastric, ovarian, testicular and lymphoma [11–17]. Furthermore, Gb3 expression correlates with the metastatic potential of human colon cancer and Gb3 enriched colon cancer cells have invasive characteristics, for which Gb3 expression is necessary and sufficient [18]. Other glycosphingolipids relevant to cancer and Fabry disease include sphingosine-1-phosphate (S1P) and Lyso-Gb3, both of which promote cell proliferation and have been found at higher levels in the plasma of male patients compared to the plasma of controls [5, 19, 20].

The incidence of cancer in patients with Gaucher disease, an LSD in which there is accumulation of the GSL glucocerebroside due to deficiency of glucocerebrosidase, has been reported [21–24]. The evidence is most persuasive of an increased risk of haematological cancer, especially multiple myeloma [21–25]. However, only a handful of case reports have been published describing cancer in individuals with Fabry disease and there has been no systematic study of the relative incidence of cancer in the context of Fabry disease compared to the general population [26–30].

In this study, we use retrospective data from hospital notes and patient questionnaires to review the incidence of cases of cancer and benign lesions within a large single centre patient cohort.

## Methods

### Patients and data collection

Adult patients (age > 18 years) who attended the Royal Free Hospital Lysosomal Storage Disorder Unit between 2012 and 2016 were eligible for inclusion in the study. Notes of patients who had consented to the retrospective database were reviewed. Additionally, a questionnaire regarding the incidence of cancer in patients and families was administered. The questionnaire had received ethical approval and all patients returning the questionnaire consented to do so. Two hundred sixty one patients were included; 11 patients were excluded from the analysis because data were insufficient, the patient was lost to follow-up before 2012 or no questionnaire had been returned.

Data collection included: gender, date of birth, treatment status, occurrence of cancer since birth (with year of occurrence) and occurrence of benign lesion since birth (with year of occurrence). Benign lesions included precancerous lesions (a histological lesion that, with time, has an increased risk of developing into cancer, for example cervical intraepithelial neoplasm), proliferative lesions (a benign tumour that does not have metastatic potential, such as a meningioma, but can cause complications e.g. due to its space occupying effect) and other lesions (such as a cholesteatoma, which is an abnormal collection of keratin).

### Analysis of results and statistical methods

In order to compare cancer incidence in the general population and Fabry population, cancer incidence rates for both cohorts were calculated. The cancer incidence rate for a cohort is defined as [31]

$$ I\equiv \mathrm{in}\mathrm{cidence}\kern0.17em \mathrm{rate}\kern0.17em \mathrm{in}\kern0.17em \mathrm{cohort}=\frac{\mathrm{total}\kern0.17em \mathrm{number}\kern0.17em \mathrm{of}\kern0.17em \mathrm{cases}\kern0.17em \mathrm{in}\kern0.17em \mathrm{the}\kern0.17em \mathrm{time}\kern0.17em \mathrm{period}}{\mathrm{total}\kern0.17em \mathrm{person}\kern0.17em \mathrm{years}\;\mathrm{at}\;\mathrm{risk}\kern0.17em \mathrm{during}\kern0.17em \mathrm{the}\kern0.17em \mathrm{time}\kern0.17em \mathrm{period}}. $$

Let *P* be the cohort population, *n*_*i*_ the number of new cases in year *i*, and *Y* the number of years in the study period. The total number of cases (*N*) during the study period is given by

$$ N=\sum_{i=1}^Y{n}_i $$

and the incidence rate can be expressed as

$$ I=\frac{N}{Y\times \left(P-N\right)+\sum_{i=1}^Y\left(i-0.5\right)\times {n}_i}. $$

Data on cases of cancer in the general population was acquired from publicly available cancer registration data (available from The Office for National Statistics website). Specifically, we used registrations of newly diagnosed cases of cancer in England between 1995 and 2014 inclusive as this was the most comprehensive data. Therefore, we used a 20-year study period from 1995 to 2014 as the primary analysis and so the incidence rate can be written as

$$ I=\frac{N}{20\times \left(P-N\right)+\sum_{i=1995}^{2014}\left(i-1995+0.5\right)\times {n}_i}. $$

The figure used for the population of England was the 2005 mid-year estimate taken from The Office of National Statistics data (50,606,000 individuals).

The cancer incidence rate for the Fabry cohort and general population cohort were compared as an incidence rate ratio [31]

$$ IR= incidence rate ratio=\frac{I_{Fabry}}{I_{Gen\; pop}}. $$

The standard deviation of log(*IR)* was calculated using

$$ SD\left[\mathrm{In}(IR)\right]={\left(\frac{1}{N_{Fabry}}+\frac{1}{N_{Gen\; pop}}\right)}^{0.5} $$

and the lower and upper limits of the rate ratio calculated using

$$ \underline{IR.}\overline{IR}=\exp \Big\{\mathrm{In}(IR)\pm 1.96 SD\left[\mathrm{In}(IR)\right]\cdot $$

The method above excludes cancer cases pre-1995 and post-2014. In the Fabry cohort there were 2 cancer diagnoses pre-1995 and 5 cancer diagnoses post-2014. We therefore decided to also perform the calculations using a 22-year study period from 1995 to 2016. To do this we estimated cancer incidence in the general population in 2015 and 2016 as the same as in 2014.

### Administrative requirements

Ethics approval was gained from University College London (UCL) and the Integrated Research Application System (IRAS). Patient information was kept confidential and managed in accordance with trust data protection guidance (which incorporates the Data Protection Act of 1998).

## Results

### Characteristics of the study population

The case notes and/or questionnaire data of 261 adult patients who attended the Royal Free Hospital Lysosomal Storage Disorder unit between 2012 and 2016 were included in the data analysis. Of these 163 (62%) were female and 98 (38%) were male. The median age was 53 years, with a lower quartile age of 41 years and an upper quartile age of 64 years. The majority of patients were Caucasian. Eighty four male patients (86%) and 80 female patients (49%) were receiving enzyme replacement therapy (ERT). All patients had a confirmed genetic diagnosis of Fabry disease.

### Characteristics of the general UK population

As we will go on to compare the Fabry population to the general population, it is worth briefly describing some features of the UK population which have relevance to cancer incidence. Whilst almost all of the Fabry patients are Caucasian the UK population is more diverse, with 86% of the population identifying with a White ethnic group in the 2011 Census (Office for National Statistics). The median age of the UK population in 2014 was 40 years, with a lower quartile age of 21 years and an upper quartile age of 58 years (Office for National Statistics).

### Characteristics of patients identified with cancer

Twenty-five patients (10%) had a previous or current diagnosis of cancer (Table 1). This was comprised of 17 females and 8 males. The most common cancer in females was breast cancer (7 cases) and in males was renal cell carcinoma (2 cases). Four patients were diagnosed with melanoma and 5 patients were diagnosed with urological malignancies (1 female with bladder cancer, 1 male with ureteric cancer, 1 female with renal cancer and 2 males with renal cancer). One female and 1 male were diagnosed with 2 separate malignancies; patient 9 had breast cancer and melanoma and patient 25 had renal cell carcinoma (clear cell) and prostate adenocarcinoma.

**Table 1:** Cancer cases in the Fabry cohort

Patient number	Gender	Year of birth	Year of cancer diagnosis	Cancer Type	Status	ERT	Smoking	Family history of cancer (type)
1	Female	1982	2016	Colon	Alive	No	Unknown	Father (unknown)
2	Female	1963	1999	Melanoma	Alive	Yes	No	Mother (bowel)
3	Female	1962	2007	Bowel	Alive	Yes	No	No
4	Female	1961	2000	Melanoma	Alive	No	No	No
5	Female	1960	2004	Breast	Alive	Yes	No	Unknown
6	Female	1953	2014	Breast	Alive	Yes	Yes	Unknown
7	Female	1952	2008	Breast	Alive	Yes	Yes	No
8	Female	1952	2005	Breast	Alive	No	No	Paternal grandmother (unknown)
9	Female	1947	1987 and 2006	Melanoma, breast	Alive	No	Unknown	No
10	Female	1945	2004	Breast	Alive	No	No	No
11	Female	1939	Unknown	Basal cell	Alive	Yes	No	No
12	Female	1936	2001	Breast	Alive	No	No	No
13	Female	1935	2016	Lung	Alive	Yes	Yes	No
14	Female	1934	1998	Lymphoma	Alive	No	No	Mother (renal)
15	Female	1932	1999	Colon	Alive	No	No	No
16	Female	1927	2006	Bladder	Lost to follow-up	No	No	Mother (liver)
17	Female	Unknown	2000	Renal	Unknown	Unknown	Unknown	Unknown
18	Male	1982	2015	Renal	Deceased	No	Unknown	Unknown
19	Male	1956	1981	Testicular	Alive	Yes	No	Father (eyelid)
20	Male	1953	2010	Basal cell	Alive	Yes	No	No
21	Male	1947	2002	Melanoma	Alive	Yes	Yes	No
22	Male	1944	2010	Mesothelioma	Deceased	Yes	No	No
23	Male	1940	2016	Left ureteric transitional cell	Alive	Unknown	Unknown	Unknown
24	Male	1940	2016	Chronic lymphocytic leukaemia	Alive	No	Ex-smoker	No
25	Male	1930	2008	Renal cell and prostate	Alive	Yes	No	No

### Comparison with general population cancer incidence rates

Using publicly available data from The Office for National Statistics the all-cancer (excluding non-melanoma skin cancer) incidence rate for the general population (using the study period 1995 to 2014) was 519 new cases per 100,000 per year. To calculate an all-cancer (excluding non-melanoma skin cancer) incidence rate for the Fabry population that was comparable to the above we had to exclude 7 individuals from the 25 with a current/past diagnosis of cancer. This included 2 patients with basal cell carcinoma (patients 11 and 20), 2 patients who had been diagnosed before 1994 (patients 9 and 19) and 5 patients who had been diagnosed after 2014 (patients 1, 13, 18 and 23 and 24). The all-cancer (excluding non-melanoma skin cancer) incidence rate of the Fabry population was 316 per 100,000 per year. The incidence rate ratio of the Fabry population compared to the general population was 0.61 (95% confidence interval 0.37 to 0.99).

The above approach has the disadvantage of excluding 7 of the 25 individuals with cancer. Therefore, we also compared cancer in the Fabry population and general population over longer study period, namely 1994 to 2016. To do this we made the assumption that cancer incidence in the general population in both 2015 and 2016 was the same as in 2014. This meant only 4 patients were excluded from the analysis. The cancer incidence rate for the general population became 531 per 100,000 per year and the cancer incidence rate in the Fabry population was 379 per 100,000 per year. The incidence rate ratio of the Fabry population compared to the general population was 0.71 (95% confidence interval 0.46 to 1.1).

As there were 5 cases of urological cancer and 4 cases of melanoma we compared the incidence rate of these specific cancers in the Fabry and general population. The incidence rate of malignant neoplasm of kidney, renal pelvis, ureter, bladder, other and unspecified urinary organs in the general population was 32 per 100,000 per year (for both the 20-year and 22-year study period). Of the 5 cases in the Fabry cohort, 2 were excluded from the 20-year analysis as they were diagnosed after 2014 (patients 18 and 23). The incidence rate of malignant neoplasm of kidney, renal pelvis, ureter, bladder, other and unspecified urinary organs in the Fabry population was 58 per 100,000 per year (1995 to 2014) and the incidence rate ratio of the Fabry population compared to the general population was 1.8 (95% confidence interval 0.58 to 5.6). With the 22-year study period, the incidence rate in the Fabry cohort was 88 per 100,000 per year and the incidence rate of the Fabry population compared to the general population was 2.7 (95% confidence interval 1.1 to 6.5). Given the significant effects of Fabry disease on the kidney parenchyma we also repeated the analysis for incidence rate of malignant neoplasm of the kidney, except renal pelvis. There were 3 cases of renal carcinoma in the Fabry cohort; 2 were included in the 20-year study period (as patient 18 was diagnosed in 2015) and all 3 were included in the 22-year study period. The incidence rate of malignant neoplasm of the kidney (except pelvis) in the general population was 12 per 100,000 per year (for both the 20-year and 22-year study periods). The incidence rate in the Fabry population was 38 per 100,000 per year for the 20-year study period, giving an incidence rate ratio of the Fabry population compared to the general population of 3.3 (95% confidence interval 0.83 to 13). In the 22-year study period, the incidence rate in the Fabry population was 52 per 100,000 per year, giving an incidence rate ratio of the Fabry population compared to the general population of 4.3 (95% confidence interval 1.4 to 13).

The malignant melanoma incidence rate in the general population is 16 per 100,000 per year (study period 1995 to 2014). One of the 4 melanoma cases in the Fabry cohort was excluded from the analysis as she had been diagnosed in 1987 (patient 9). The incidence rate of malignant melanoma in the Fabry population was 58 per 100,000 per year (study period 1995 to 2014). The incidence rate ratio of the Fabry population compared to the general population was 3.6 (95% confidence interval 1.2 to 11). In the Fabry cohort there were no melanoma diagnoses post 2014. When the calculations were repeated with the extended study period (1994 to 2016), the incidence rate in the general population was 17 per 100,000 per year, the incidence rate in the Fabry cohort was 53 per 100,000 per year and the incidence rate ratio was 3.1 (95% confidence interval 0.99 to 9.5).

### Characteristics of patients identified with benign lesions

Twenty-four patients in the cohort (9%), 17 females and 7 males, had one or more diagnoses of benign lesions (Table 2). The most common were growths in neurological tissues (5 cases), colon polyps (5 cases), benign breast lesions (4 cases), atypical moles (3 cases), renal lesions (2 cases) and cervical intraepithelial neoplasm (2 cases).

**Table 2:** Cases of benign lesions in the Fabry cohort

Patient number	Gender	Year of birth	Year of benign lesion diagnosis	Benign lesion	Status	ERT	Smoking	Family history of cancer (type)
26	Female	1986	Unknown	Benign acoustic neuroma	Alive	No	No	Unknown
27	Female	1984	2004	Cervical intraepithelial neoplasia	Alive	Yes	No	Maternal grandmother (unknown)
1	Female	1982	Unknown	Atypical mole	Alive	No	Unknown	
28	Female	1979	2011	Small falx meningioma	Lost to follow-up	No	No	Father (unknown)
29	Female	1976	Unknown	Cervical intraepithelial neoplasia	Alive	No	Unknown	No
30	Female	1975	2005	Fibroadenoma of breast	Alive	No	Yes	No
31	Female	1972	2007	Atypical mole	Alive	No	No	No
32	Female	1969	1987	Craniopharyngioma	Alive	No	No	No
33	Female	1966	2010	Prolactinoma	Alive	No	Yes	Unknown
34	Female	1962	2001 and 2003	Fibroadenoma of breast, lipoma	Alive	Yes	Yes	No
4	Female	1961	2001	Atypical mole	Alive	No	No	No
35	Female	1961	2009	Breast benign lesion	Alive	Yes	No	Unknown
36	Female	1959	Unknown	Colon polyp	Lost to follow-up	Yes	Yes	No
37	Female	1958	2016	Meningioma	Alive	Yes	Unknown	Unknown
38	Female	1943	1990, 1995 and 2011	Colon polyps ×2, nodular lesion left adrenal	Alive	Yes	Ex-smoker	No
13	Female	1935	2001	Colon polyp	Alive	Yes	Ex-smoker	No
16	Female	1927	1972	Benign breast neoplasm	Lost to follow-up	No	No	Mother (Liver)
20	Male	1953	1974	Left ear cholesteatoma	Alive	Yes	No	No
39	Male	1951	2006	Gastric dysplasia	Lost to follow-up	Yes	No	Unknown
40	Male	1949	2005	Colon polyp	Alive	Yes	Yes	Unknown
41	Male	1939	2010	Adrenal adenoma	Alive	Yes	Ex-smoker	No
42	Male	1935	2010	Monoclonnal gammopathy of undetermined significance (MGUS)	Lost to follow-up	Yes	No	No
43	Male	1933	2009	Neurofibroma, haemangioma	Deceased	Yes	Ex-smoker	No
44	Male	1931	2001 and 2006	Colonic polyps	Deceased	Yes	Ex-smoker	No

Three patients were diagnosed with two benign lesions; patient 34 had fibroadenoma of the breast and lipoma, patient 38 had colon polyps and renal nodule and patient 43 had neurofibroma and haemangioma.

Five patients were diagnosed with both a cancer and a benign lesion; patient 1 had atypical mole and colon cancer, patient 4 had atypical mole and melanoma, patient 13 had colon polyp and lung cancer, patient 16 had benign breast neoplasm and bladder carcinoma, and patient 20 had left ear cholesteatoma followed by basal cell carcinoma.

### Comparison with general population meningioma incidence rates

Benign tumours and precancerous lesions in the general population are not systematically recorded in the manner of malignancies and therefore it was not possible to compare our Fabry cohort to the general population except in the case of benign neoplasm of meninges.

There were 2 diagnoses of benign neoplasm of the meninges in the Fabry cohort. For the 20-year study period (1995 to 2004) the incidence rate of benign neoplasm of meninges in the general population was 2.8 per 100,000 per year. One diagnosis of benign meningioma occurred between 1995 and 2014 and the incidence rate of benign neoplasm of meninges in the Fabry population over this period was 19 per 100,000 per year. The incidence rate ratio of the Fabry population compared to the general population was 6.8 (95% confidence interval 0.96 to 49).

For the 22-year study period (1995 to 2016) the incidence in the general population was 2.9 per 100,000 per year and the Fabry population was 35 per 100,000 per year. The incidence rate ratio was 12 (95% confidence interval 3.0 to 48).

## Discussion

### Cancer in patients with Fabry disease

#### All cancer (excluding non-melanoma skin cancer) incidence

Our study identified 25 cases of cancer in the Fabry cohort. When compared to the general population there appeared to be a borderline-significant reduction in risk of cancer in the Fabry patients with an incidence rate ratio of 0.61 (95% confidence interval 0.37 to 0.99) in the 1995–2014 analysis and an incidence rate ratio of 0.71 (95% confidence interval 0.46 to 1.1) in the 1995–2016 analysis.

Reliable comparison of our small Fabry patient cohort to the general population is challenging due to multiple confounding factors which may explain the difference in cancer risk. The demographics of the two populations also differ with a median age of around 41 years in the general population and 53 years in the Fabry population. The Fabry population is followed up yearly in clinic and frequent interaction with health care services may lead to a healthier lifestyle and earlier risk factor intervention. For example, Fabry patients have their smoking status, weight, blood pressure, cholesterol and basic bloods monitored at each clinic visit. In addition, frequent medical reviews may help to identify precancerous lesions before they become malignant.

There may also be ascertainment bias and cases of cancer in the cohort may be missing. In this study, a high proportion of the cancer cases were recent diagnoses, suggesting recording of malignancies could have improved and older cases may be missing from the dataset. Similarly, a study of malignancy in Gaucher disease described a lower risk of solid tumours (when compared to the general population) and ascertainment bias was recognised as a potential factor [25].

However, it is possible that alteration in the sphingolipid profile could impact on cancer development. Fabry disease affects endothelial cells and results in abnormal tissue perfusion; since angiogenesis is a requirement for tumour growth, neoplasia in Fabry disease may be compromised by chronic ischemia [32]. In addition, a higher baseline apoptotic rate may be protective against malignant transformation [33].

#### Urological cancers

There were 5 cases of urological cancer in the Fabry cohort. Two were excluded in the 20-year analysis and there was an incidence rate ratio of 1.8 (95% confidence interval 0.58 to 5.6). However, in the 22-year analysis all 5 cases could be included and this led to an incidence rate ratio of 2.7 (95% confidence interval 1.1 to 6.5). Considering renal cancer alone, there were 2 cases in the 20-year analysis with an incidence rate ratio of 3.3 (95% confidence interval 0.83 to 13) and 3 cases in the 22-year analysis with an incidence rate ratio of 4.3 (95% confidence interval 1.4 to 13). Therefore, this may represent an excess of urological malignancy, and also specifically renal cancers, in the Fabry cohort.

This is of particular relevance because the kidneys can be severely affected in Fabry disease, with proteinuria and end stage renal function possible if the condition is untreated [6]. Chronic kidney damage and inflammation may be carcinogenic, as could exposure to excess sphingolipids.

Patients with Fabry disease have more frequent investigation compared to the general population, including blood tests, urine analysis and renal ultrasounds. This might lead to an increase in the detection of renal carcinoma, however the natural history of renal cancer would suggest that similar cases in the general population would not go undetected indefinitely [34].

There is one case of testicular cancer in the Fabry cohort. This is of particular interest because azoospermia and infertility are common in ‘classic’ Fabry males and, should more cases of testicular cancer emerge, the mechanisms behind the defects in testicular function may merit further investigation [35].

#### Malignant melanoma

We also detected a possible excess of melanoma in the Fabry cohort, with an incidence rate ratio of 3.6 (95% confidence interval 1.2 to 11) in the 20-year analysis and an incidence rate ratio of 3.1 (95% confidence interval 0.99 to 9.5) in the 22-year period.

Skin changes, such as angiokeratoma and telangiectasia, are common manifestations of Fabry disease and the presence of cutaneous vascular lesions is correlated with the severity of systemic manifestations [36]. It is possible that abnormalities in the skin could be linked to the increased rates of melanoma in Fabry patients.

However, the majority of patients in the Fabry cohort are Caucasian (which confers a higher risk of melanoma) and the national data is likely to reflect the greater variation of ethnicities and skin types in the general population. We were also unable to collect further data regarding risk factors such as sun exposure. Skin is reviewed frequently in clinic and many patients will have formal dermatology check-ups. Higher detection rates may explain this apparent excess but, as with urological cancer, malignant melanoma inevitably tends to self-declare.

### Benign lesions in patients with Fabry disease

Twenty-four patients in the Fabry cohort had a documented benign lesion. Whilst benign breast lesions, colon polyps and atypical moles are frequently seen in clinical practice, benign growths of neurological tissues were the most common benign lesion in Fabry patients (5 cases). There is a single case report of 3 Fabry patients with meningiomas [37].

Benign growths of neurological tissue may be over represented in our series; given the high stroke risk in Fabry, these patients have routine brain MRI scans and thus some meningiomas could be incidental findings. However, it is possible that vascular abnormalities in Fabry disease contribute to benign brain tumour growth.

## Conclusion

Overall our data suggests that patients with Fabry disease do not seem to be at highly increased risk of cancer development. However, there may be an increased incidence of melanoma, urological cancers and benign meningioma in Fabry patients. This could be due stimulation by lyso-lipids, disease-related inflammation and vascular abnormalities. Limitations of our study include recall bias and ascertainment bias (due to increased monitoring frequency of the population). Further studies should address these problems prospectively, in a larger cohort of patients.

## Abbreviations

AGAL A: *α*-galactosidase A
ERT: Enzyme replacement therapy
Gb3: Globotriaosylceramide
GSL: Glycosphingolipids
LSD: Lysosomal storage disorder
Lyso-Gb3: Globotriaosylsphingosine
